# Improving the Dispersibility of TiO_2_ in the Colloidal System Using Trifunctional Spherosilicates

**DOI:** 10.3390/ma16041442

**Published:** 2023-02-08

**Authors:** Bogna Sztorch, Krzysztof Nowak, Miłosz Frydrych, Julia Leśniewska, Klaudia Krysiak, Robert E. Przekop, Anna Olejnik

**Affiliations:** 1 Centre for Advanced Technology, Adam Mickiewicz University Poznan, Uniwersytetu Poznańskiego 10, 61-614 Poznań, Poland; 2 Faculty of Chemistry, Adam Mickiewicz University Poznan, Uniwersytetu Poznańskiego 8, 61-614 Poznań, Poland

**Keywords:** POSS, silsesquioxanes, emulsion, surface modification, titanium(IV) oxide, UV filter

## Abstract

Titanium dioxide is a commonly used ingredient in cosmetics acting as a thickening agent and inorganic UV filter. However, TiO_2_ is difficult to disperse, which causes problems in spreading the formulations. The solution to this problem is to modify the titanium dioxide surface to change its properties by creation of the new type of hybrid inorganic–organic UV filter. Therefore, this study aimed to functionalize titanium dioxide with organosilicon compounds and determine how this modification will affect the dispersibility of TiO_2_ in the colloidal system and the stability of emulsions. First, the functionalized octaspherosilicates were obtained and characterized. Next, the synthesized compounds were applied as modifiers for titanium dioxide and were analyzed by FT-IR, UV-Vis, and laser diffraction. Furthermore, the hydrophilic–hydrophobic character was assessed by measuring the contact angle. The new materials were introduced into emulsions and the formulations were analyzed in terms of particle size distribution and stability by multiple light scattering. It was found that the modification of titanium dioxide with spherosilicates significantly improved both the stability of emulsion and the dispersibility of novel materials in the colloidal system compared to nonmodified TiO_2_. The covalent binding of the modifier with the titanium dioxide had an impact on the stability of the emulsion.

## 1. Introduction

Titanium dioxide (TiO_2_) is a commonly used ingredient in cosmetic products such as creams, powders, toothpaste, shampoos, soaps, nail polish, and eye shadows [1]. It acts as a thickening agent and a physical filter protecting against ultraviolet radiation [2,3,4]. Titanium dioxide naturally exists in different crystalline forms such as anatase, brookite, and rutile [5,6,7]. It was proved that rutile was more stable than anatase and brookite at room temperature [8]. Each form has different properties and applications. Titanium dioxide also exhibits high photocatalytic activity [9,10,11], which is associated with the generation of reactive oxygen species that may adversely affect the skin, accelerating the aging process [12,13]. Moreover, it is difficult to disperse TiO_2_ while preparing the formulations. Therefore, it is sometimes problematic to spread cosmetic products on the skin. Furthermore, it leaves white spots on its surface. The solution to this problem may be to modify the titanium dioxide, which enables to change its properties. So far, the surface of TiO_2_ has been functionalized with 3-aminopropyltriethoxysilane and N-2-(aminoethyl)-3-aminopropyltrimethoxysilane to be used in the adsorption processes of different dyes [14]. In other studies, the surface of titanium dioxide was treated with methacryloxypropylyrimethoxysilane to reduce the photocatalytic activity [15]. In turn, Yao et al. modified TiO_2_ with a silane coupling agent to change the surface properties from hydrophilic to hydrophobic [16]. The variation of surface nature was also observed when titanium dioxide was functionalized with sodium stearate and sodium oleate [17]. Recent studies have shown that the use of functionalized silsesquioxanes as modifiers of TiO_2_ increased its hydrophobicity and improved covering and dispersing properties [18].

Silsesquioxanes belong to the class of compounds that are hybrid systems, most often with a cubic structure and the general formula (RSiO1.5)n where R=H, alkyl, aryl, or their derivatives where n = 6, 8, 10, 12 [19]. An inorganic core and organic functional groups R attached to each of the eight corners of the cage can be distinguished in their structure. Silsesquioxanes can change the physicochemical properties of materials, such as thermal resistance [20], surface energy, and water contact angle [21] or they can improve dispersion [18] or even act as compatibilizers [22]. The factors influencing the dispersion capacity of particles in the system and the strength of their interaction with the modifier or matrix include particle size, specific surface area/porosity, chemical composition, and the presence/absence of surface functional groups in the case of fillers and pigments [23]. One of the most commonly used methods to improve dispersion is surface silanization, which reduces the ionic character of their surface and improves the wetting effect of the surface with the polymer. The modification of TiO_2_-SiO_2_ or SiO_2_ with the use of silsesquioxanes has been reported by Nowacka et al. [24] and Szwarc et al. [25], respectively.

The aim of this study was to functionalize titanium dioxide with organosilicon compounds (spherosilicates) and determine how this modification will affect the dispersibility of TiO_2_ in the colloidal system as well as the stability of emulsions. The trifunctional spherosilicates were selected because each introduced group exhibited specific functionality. Vinyl trimethoxysilane was used as a substrate to attach the anchoring group, derivative of cinnamate and eugenol were applied as the UV absorbing agent, whereas hexene and octene were used as the hydrophobic groups. Diethylene glycol monovinyl ether and allyl glycidyl ether were applied as substrates in the hydrosilylation reaction to introduce hydrophilic groups.

## 2. Materials and Methods

### 2.1. Synthesis of the Trifunctional Octasubstituted Spherosilicates

All spherosilicate derivatives were obtained by the hydrosilylation process, using commercially available olefins. The substrates applied to carry out the reactions are presented in Table 1. A precursor for the synthesis was obtained according to a literature procedure [26]. A total of 30 g of octaspherosilicate was placed in a three-neck, round-bottom flask (Glassco, Ambala, India), next, 250 mL of toluene and the mixture of:

(1) allyl cinnamate (22.20 g), hexene (7.45 g), and diethylene glycol monovinyl ether (3.90 g) in molar ratio 4:3:1
(2) allyl cinnamate (16.65 g), hexene (9.93 g), and allyl glycidyl ether (3.37 g) in molar ratio 3:4:1
(3) allyl cinnamate (22.20 g), octene (9.93 g), and vinyltrimethoxysilane (4.37 g) in molar ratio 4:3:1
(4) eugenol (14.53 g), octene (9.93 g), and vinyltrimethoxysilane (8.74 g) in molar ratio 3:3:2 was added.

**Scheme 1:**
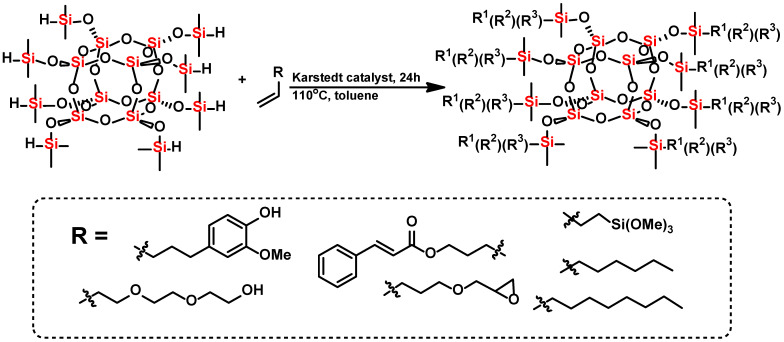
Hydrosilylation of olefins with octaspherosilicate.

The reactions’ mixture was constantly stirred and heated to 60 °C (then 30 µL of Karstedt’s catalyst solution in xylene was added). The process was carried out for 24–48 h under reflux according to Scheme 1. The progress of the reaction was monitored by FT-IR-ATR analysis (Thermo Fisher Scientific, Waltham, MA, USA) until the Si-H bands’ disappearance (at 2141 cm^−1^ and 889 cm^−1^—stretching and bending vibrations, respectively).

The compound conversion as well as the purity of the products were determined by ^1^H, ^13^C, and ^29^Si NMR spectroscopy analysis.

### 2.2. Modification of Titanium Dioxide with Octaspherosilicate Derivatives

The procedure of mixing TiO_2_ with the selected organosilicon compound was carried out in accordance with our previous work [18]. In the modification process, all systems were obtained comprising of 1% *w*/*w* modifiers content in the dry TiO_2_.

### 2.3. Preparation of Emulsions with Modified Titanium Dioxide

The water phase and an oil phase were prepared separately with the compositions shown in the Table 2. Both phases were heated to 70 °C until all ingredients were dissolved. Modified TiO_2_ was added to the oil phase and mixed. Afterwards, both phases were combined and homogenized on a SilentCrusher M homogenizer (Heidolph, Schwabach, Germany) for 4 min. at 16,000 rpm. After cooling the emulsion to 30 °C, phenoxyethanol was added as preservative. Emulsions were prepared with each modified titanium dioxide and with the nonmodified one as the reference system.

### 2.4. Analytical Methods

^1^H, ^13^C, and ^29^Si Nuclear Magnetic Resonance (NMR) spectra were recorded at 25 °C on a Bruker Ascend 400 and Ultra Shield 300 spectrometers (Bruker, Billerica, MA, USA) using CDCl_3_ as a solvent. Chemical shifts were reported in ppm with reference to the residual solvent (CHCl_3_) peaks for ^1^H.

Density measurements of octaspherosilicate derivatives were performed three times on stationary densimeter KEM (Kyoto Electronics Manufacturing, Kyoto, Japan) at a temperature of 20 °C.

Water contact angle (WCA) analyses were performed five times by the sessile drop technique at room temperature and atmospheric pressure with a Krüss DSA100 goniometer (Krüss, Hamburg, Germany). Three independent measurements were taken for each sample, each with a 5 µL water drop.

UV spectra of octaspherosilicate derivatives were recorded using a UV spectrophotometer Cary 60 (Agilent Technologies, Santa Clara, CA, USA). Samples were dissolved in n-hexane. Solutions with the following concentrations were prepared: 1.0335 × 10^−5^ mol/L for SSQ1, SSQ2, SSQ3 and 2.1008 × 10^−5^ mol/L for SSQ4. Solid state UV-Vis spectra in reflectance mode were recorded by a UV-Vis spectrophotometer (Cary 60, Agilent) equipped with the solid sample holder.

The FT-IR spectra of modified and nonmodified titanium dioxide were recorded on a Nicolet iS50 spectrometer (Thermo Fisher Scientific, Waltham, MA, USA). For this purpose, KBr pellets were made (200 mg KBr + 2 mg of tested titanium dioxide). Pellets were put into spectrometer and transmission measurements were made.

The refractive index was measured three times using a refractometer InsMark IR120. Particle size distribution of modified and nonmodified titanium dioxide as well as emulsions was determined by a Mastersizer 3000 (Malvern Instruments Co. Ltd., Malvern, UK) using the laser diffraction method. The analyses were conducted in distilled water at room temperature. Five independent measurements were taken for each sample.

The zeta potential of titanium dioxide nonmodified and modified with SSQs was determined in distilled water by a Zetasizer Nano ZS (Malvern Instruments Ltd., Malvern, UK).

A digital light microscope Keyence VHX 7000 (Keyence International, Mechelen, Belgium) with 100- x1000 VH-Z100T lens was used to examine the pigment dispersion in the emulsion. All images were recorded with a VHX 7020 camera.

The stability of the emulsion was measured using a Multiscan MS 20 (DataPhysics, Instruments GmbH, Filderstadt, Germany). The backscattering profiles were determined and presented as graphs versus sample height. All emulsions were analyzed for 31 days at a certain time at 25 °C.

## 3. Results and Discussion

### 3.1. Characterization of Organosilicon Modifiers

**Figure 1:**
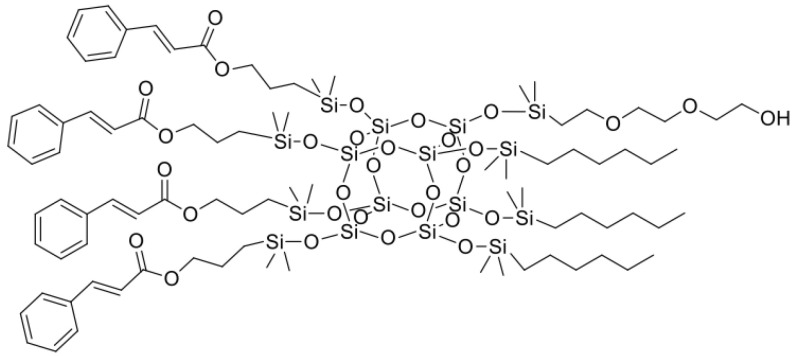
The chemical structure of SSQ1.

**Figure 2:**
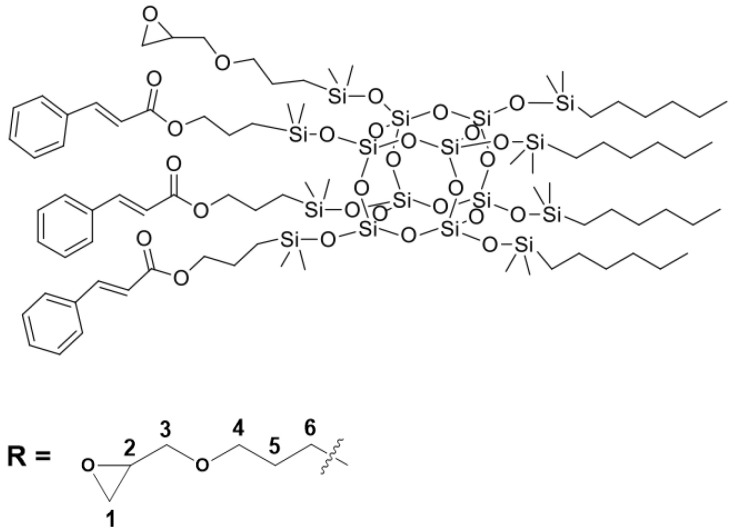
The chemical structure of SSQ2.

**Figure 3:**
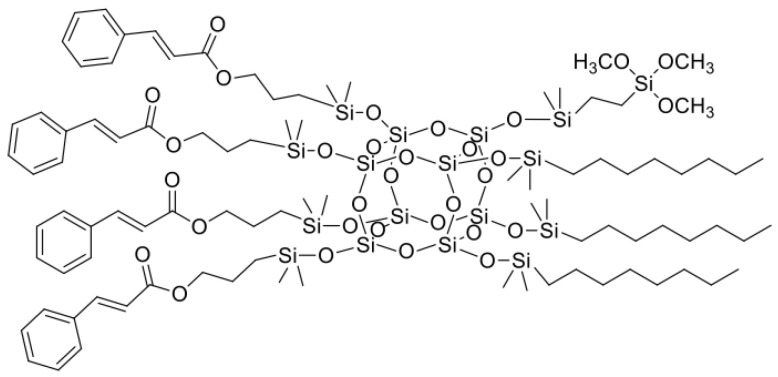
The chemical structure of SSQ3.

**Figure 4:**
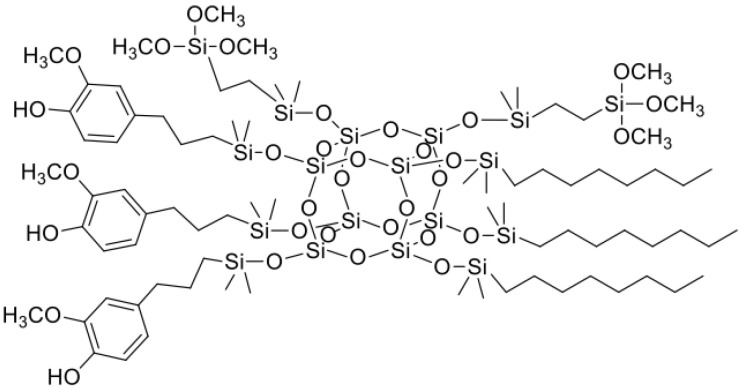
The chemical structure of SSQ4.

All organosilicon derivatives were obtained by the hydrosilylation reaction of octahydrospherosilicate and commercially available olefins with groups containing unsaturated bonds capable of absorbing UV radiation, as well as non-polar hydrophobic groups and polar groups such as oxirane group, trimethoxysilyl, or diethylene glycol ether. In addition, the trimethoxysilyl group was used to create weak chemical bonds between alkoxyl groups of SSQ and the hydroxyl groups located on the surface of titanium dioxide. Different derivatives containing three types of functional groups in the octaspherosilicate cage were synthesized. Their chemical structures are presented in Figure 1, Figure 2, Figure 3 and Figure 4. All compounds were obtained with high yield (>95%) and full conversion (>99%), which confirms the efficiency of the hydrosilylation reaction in obtaining functionalized organosilicon compounds. The conversion of the substrates and the purity of the products were determined by ^1^H NMR spectroscopy.

^1^H NMR (600 MHz, CDCl_3_) δ (ppm): 7.70 (m, 4H, CH=CH-Ph), 7.54–7.39 (m, 20 H, Ph), 6.48 (m, 4 H, CH=CH-Ph), 4.20 (m, CH_2_- CH_2_- CH_2_-O), 4.02–3.63 (m, 10H, O-CH_2_- CH_2_-O) 2.96, 2.65,1.78 (m, CH_2_- CH_2_- CH_2_-O), 1.34–1.30 (m, 24 H, CH_2_-(CH_2_)_4_-CH_3_), 1.06–0.97 (m, 2H, Si-CH_2_-CH_2_-O) 0.90 (m, CH_2_-CH_3_), 0.68 (m, Si-CH_2_-CH_2_-CH_2_) 0.63 (m, CH_2_-(CH_2_)_4_-CH_3_), 0.47 (m, Si-CH_2_ backbiting product) 0.21–0.16 (m, 48H, SiMe_2_).

^13^C NMR (151 MHz, CDCl_3_) δ (ppm): 167.08 (C=O), 144.71 (Ph-CH=CH), 137.94, 134.58, 130.31, 129.14, 128.97, 128.33, 125.41, 118.36 (Ph-CH=CH), 67.02, 65.32 (O-CH_2_), 33.12, 31.70, 23.01, 22.72, 22.53, 21.57, 17.76, 14.27, 13.77, −0.20, −1.30 (SiMe_2_).

^29^Si NMR (119 MHz, CDCl_3_) δ (ppm): 13.15 (SiMe_2_), −109.01 (core).

^1^H NMR (600 MHz, CDCl_3_) δ (ppm): 7.70 (m, 3H, CH=CH-Ph), 7.53–7.39 (m, 15 H, Ph), 6.45 (m, 3 H, CH=CH-Ph) 4.19 (m, CH_2_- CH_2_- CH_2_-O), 3.68 (m, 1 H position 3), 3.45 (m, 2 H, position 4), 3.40 (m, 1 H, position 3), 3.15 (m, 1 H, position 2), 2.79 (m, 1 H, position 1), 2.61 (m, 1 H, position 1), 1.78 (m, CH_2_- CH_2_- CH_2_-O), 1.67 (m, 2 H, position 5), 1.34–1.30 (m, 32H, CH_2_-(CH_2_)_4_-CH_3_), 0.90 (m, 12H CH_2_-CH_3_), 0.69 (m, Si-CH_2_-CH_2_-CH_2_), 0.62(m, Si-CH_2_-CH_2_-), 0.46 (m, Si-CH_2_ backbiting product), 0.21–0.15 (m, 48H, SiMe_2_).

^13^C NMR (151 MHz, CDCl_3_) δ (ppm): 167.09(C=O), 145.97, 144.70 (Ph-CH=CH), 137.96, 134.60, 130.31, 129.15, 128.98, 128.34, 125.42, 118.37 (Ph-CH=CH), 74.21, 71.54, (glycidoxy group) 67.05 (O-CH_2_), 50.94 (OMe), 44.45, 33.14, 31.71, 23.03, 22.73, 22.54, 21.58, 17.76, 14.27, 13.79, −0.28, −0.37 (SiMe_2_).

^29^Si NMR (119 MHz, CDCl_3_) δ(ppm): 12.93 (SiMe_2_), −109.02 (core).

^1^H NMR (600 MHz, CDCl_3_) δ (ppm): 7.73 (m, 4 H, CH=CH-Ph), 7.53–7.39 (m, 20 H, Ph), 6.48 (m, 3 H, CH=CH-Ph), 4.19 (m, CH_2_- CH_2_- CH_2_-O), 3.59 (m, 9 H, Si-O-CH_3_), 1.78 (m, CH_2_- CH_2_- CH_2_-O), 1.28 (m, 36 H, CH_2_-(CH_2_)_6_-CH_3_), 0.90 (m, 9H CH_2_-CH_3_), 0.70 (m, Si-CH_2_-CH_2_-CH_2_-O) 0.62 (m, Si-CH_2_-CH_2_), 0.46 (m, Si-CH_2_ backbiting product), 0.21–0.16 (m, 48 H, SiMe_2_).

^13^C NMR (151 MHz, CDCl_3_) δ (ppm): 167.09(C=O), 145.98, 145.18, 144.70 (Ph-CH=CH), 137.97, 134.60, 132.42, 130.31, 128.98, 128.17, 125.42, 118.38 (Ph-CH=CH), 67.04, 65.33 (O-CH_2_), 50.67 (Si-(OMe)_3_), 33.56, 32.07, 29.45, 22.81, 22.54, 21.58, 17.78, 14.25, 13.77, 8.61, 0.43, −0.20, −0.39, −0.98 (SiMe_2_).

^29^Si NMR (119 MHz, CDCl_3_) δ (ppm):13.25 (SiMe_2_), −41.72 (Si(OMe)_3_, −108.99 (core).

^1^H NMR (600 MHz, CDCl_3_) δ (ppm): 6.82–6.67 (m, 9 H, CH_2_-Ph), 5.50 (s, 3 H, O-H), 3.87 (m, 9 H, Ph-O-CH_3_), 3.58 (m, 18 H, Si-O-CH_3_), 2.56 (m, 6 H, CH_2_-Ph), 1.64 (m, 6 H, CH_2_-CH_2_-Ph), 1.28 (m, 36 H, Si-CH_2_-(CH_2_)_6_-CH_3_) 0.89 (m, 9 H, Si-CH_2_-(CH_2_)_6_-CH_3_), 0.63 (m, 20 H, Si-CH_2_) 0.14 (m, 48 H, SiMe_2_)

^13^C NMR (151 MHz, CDCl_3_) δ (ppm): 146.20, 143.49, 137.77, 134.39, 128.95, 128.14, 125.22, 120.92, 114.05, 110.92, 55.73 (Ph-O-CH_3_), 50.48 (Si-(OMe)_3_), 39.12, 33.38, 31.89, 29.27, 25.17, 22.90, 22.62, 21.38, 17.60, 17.30, 14.05, 8.40, 0.22, −0.40, −0.42, −1.20 (SiMe_2_).

^29^Si NMR (119 MHz, CDCl_3_) δ (ppm): 12.84 (SiMe_2_), −41.59 (Si(OMe)_3_, −108.89 (core).

**Figure 5:**
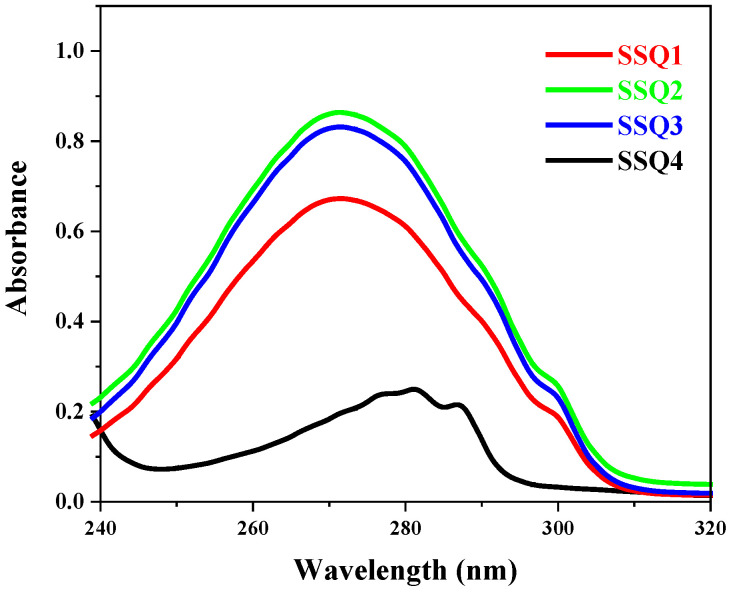
UV spectra of synthesized silsesquioxanes.

The UV spectra of spherosilicates were recorded and are depicted in Figure 5. SSQ1, SSQ2, and SSQ3 exhibit maximum absorbance at 271 nm, which can be attributed to π,π* transition with a molar absorption coefficient in the range of (6–8) × 10^4^ M^−1^ cm^−1^. The absorption band corresponds to conjugated double bonds and the aromatic ring of allyl cinnamate [27].

In turn, the absorption spectrum of SSQ4 can be attributed to a single electronic transition with an oscillation structure band with λ_max_ around 281 nm and molar absorption coefficient 1.1 × 10^4^ M^−1^ cm^−1^ (π,π* transition). Similar results were obtained by Foudah et al. [28]. The absorption band at λ_max_ at 281 nm corresponded to to the condensed benzene ring system of eugenol [29].

The density and refractive index were tested for the chemical compounds obtained (Table 3). The density value for all tested silsesquioxanes ranges from 1.093 to 1.112 g/mL, which is consistent with the data described by Joshi and Bhattacharyya [30]. Most of the volume is occupied by the functional organic groups, which are located outside the core. Typically, 5% of the volume is taken by the core [30].

### 3.2. Characterization of Modified Titanium Dioxide

**Figure 6:**
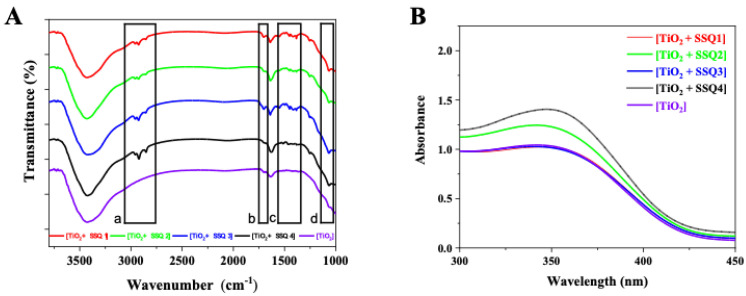
FT-IR (**A**) and UV-Vis (**B**) spectra of modified titanium dioxide.

In the FT-IR spectra of the modified titanium dioxides, in addition to the bands characteristic of TiO_2_, new signals from the bonds present in the modifiers appeared (Figure 6). All modified systems showed additional bands in the range of 3000–2700 cm^−1^ (a) and 1550–1450 cm^−1^ (c) characteristic of unsaturated aliphatic chains, originating from the cinnamate and eugenol groups, as well as signals present in the range of 1100–1000 cm^−1^ (d) indicating presence of C-O-C ether bonds. In addition, in the range of 1750–1650 cm^−1^ (b), there is a signal coming from the stretching vibrations of ketone groups contained in allyl cinnamate used in modifiers SSQ1–SSQ3. The presence of additional signals in the FT-IR spectrum confirmed the modification of the TiO_2_ surface.

In Figure 6B, the UV-Vis spectra of nonmodified and modified titanium dioxide is presented. All materials absorb light at a wavelength below 400 nm. Similar spectra of pure TiO_2_ were recorded by Zhao et al. [31]. The results proved that all new materials exhibit strong absorption in the ultraviolet region. The modification of titanium dioxide did not shift the absorption maximum to higher wavelengths.

**Figure 7:**
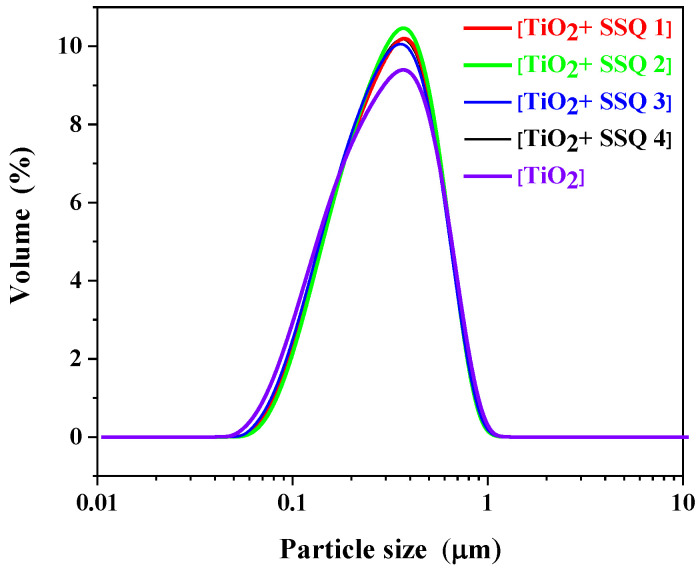
Particle size distribution of nonmodified and modified titanium dioxide.

The influence of modifying titanium dioxide with spherosilicates on particle size distribution was also tested. A monomodal particle size distribution between 0.04 and 1.00 μm for titanium dioxide was observed (Figure 7). These results are in accordance with data recorded by Song et al. [32]. For all modified materials a monomodal particle size, distribution was also detected, and no significant changes compared to pure titanium dioxide were observed.

Table 4 presents the data in the form of d (0.1), d (0.5), and d (0.9). The obtained results indicated no meaningful effect of the applied modifiers on the variation in the particle size distribution compared to TiO_2_. Table 4 also shows the results of zeta potential of samples obtained. It was proved that the nonmodified TiO_2_ suspension had a positive zeta potential value. These results are in accordance with data recorded by Ridley et al. [33] and Halimi et al. [34]. The modification of titanium dioxide with SSQs changed the surface charge from positive to negative. It should be highlighted that all suspensions can be considered as stable. Based on the literature data, the dispersion with the ζ potential value higher than ±25 mV is less prone to change their particle size [35].

**Figure 8:**
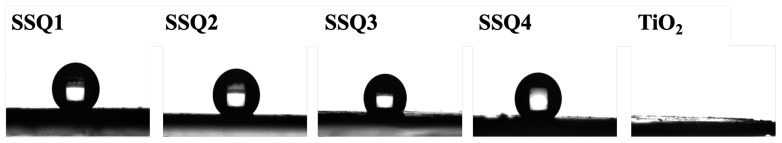
Images of water droplets during sessile drop analysis of TiO_2_+SSQ1, TiO_2_+SSQ2, TiO_2_+SSQ1, TiO_2_+SSQ1, and unmodified TiO_2_.

In order to determine the hydrophilic–hydrophobic nature of the TiO2 pigment, water contact angle analysis (WCA) was performed. The study was carried out for unmodified TiO2 as a reference sample, as well as for TiO2 with the addition of SSQ1, SSQ2, SSQ3, and SSQ4 derivatives. For the laboratory test, samples in the form of pellets (load 120 MPa for 3 min.) were prepared. The TiO2 surface has a strongly polar, superhydrophilic character; therefore, the dosed drop was completely absorbed by the tested material (Figure 8). A similar effect was observed in our previous work [22]. Modification of the TiO2 surface with organosilicon compounds containing non-polar alkyl groups, i.e., hexyl and octyl groups, and polar groups, e.g., AGE, VTMOS, or DGME changed the character of the surface. Table 5 presents the values of contact angles for individual derivatives, and in Figure 8, the results are depicted as images of water droplets during sessile drop analysis. The smallest affinity to water molecules was reported for the SSQ2 sample in which the share of allyl groups is the highest. Other derivatives also showed a strongly hydrophobic character, and the WCA values are in the range of 130–142°.

**Figure 9:**
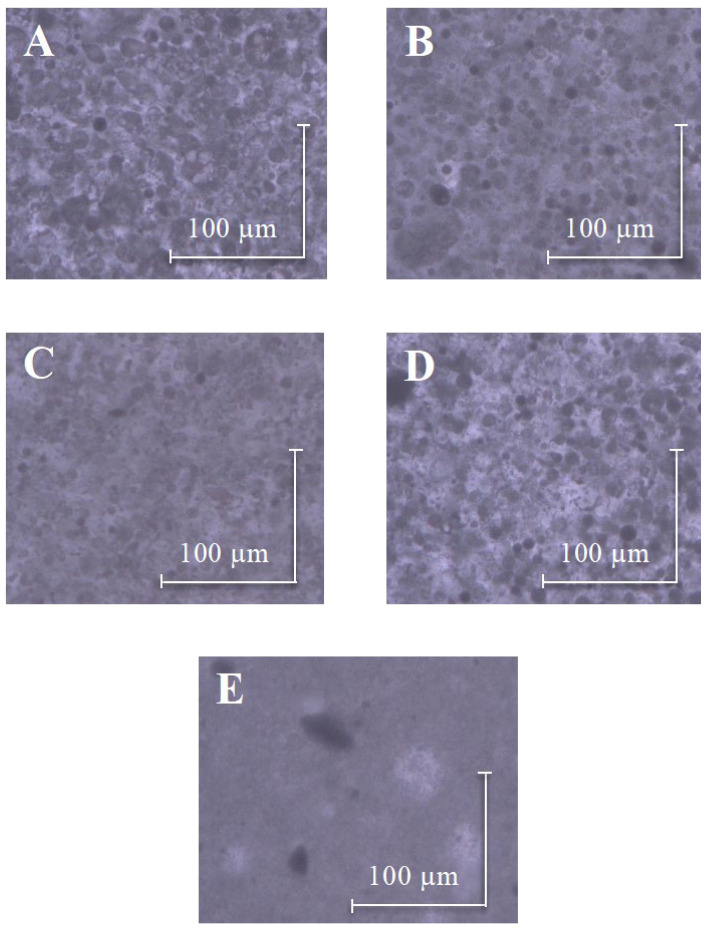
Optical microscope images of E+[TiO_2_+SSQ1] (**A**), E+[TiO_2_+SSQ2] (**B**), E+[TiO_2_+SSQ3] (**C**), E+[TiO_2_+SSQ4] (**D**), E+[TiO_2_] (**E**).

The effect of modifiers on the dispersibility of titanium dioxide in emulsions was studied by an optical microscope. In Figure 9A–D, presenting the emulsions with modified TiO_2_, much smaller droplets were observed compared to formulations containing nonmodified titanium dioxide. In the image of the emulsion with TiO_2_ (Figure 5E), large clusters of undispersed titanium dioxide were detected. Based on the obtained images, it can be concluded that the addition of spherosilicates modifiers improved the dispersion of titanium(IV) oxide in emulsions.

**Figure 10:**
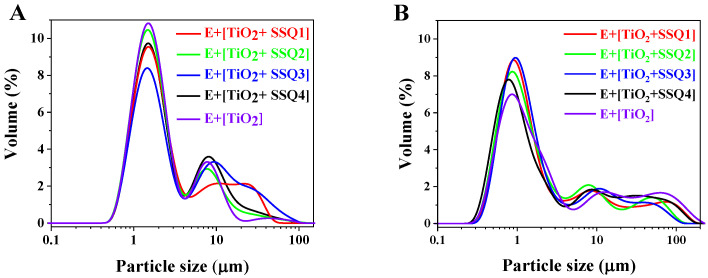
Particle size distribution of emulsions after preparation (**A**) and after 3 months (**B**).

It should be mentioned that particle size distribution might have an impact on the emulsions’ stability [36]. Therefore, all formulations were subjected to laser diffraction analyses. The results directly after the emulsions’ preparation are presented in Figure 10A. For all colloidal systems containing modified TiO_2,_ the bimodal particle size distribution was detected. The predominant peak was identified in the range of 0.7 to 8 μm. In turn, the emulsion with pure titanium dioxide had trimodal particle size distribution. It was observed in Figure 10B that after 3 months of the emulsions’ storage, the volume fraction of predominant peak decreased, and the particle distribution shifted towards a larger size.

Based on the results presented in Table 6, it could be concluded that the highest changes in particle size distribution after 3 months were observed for emulsions containing nonmodified titanium dioxide.

**Figure 11:**
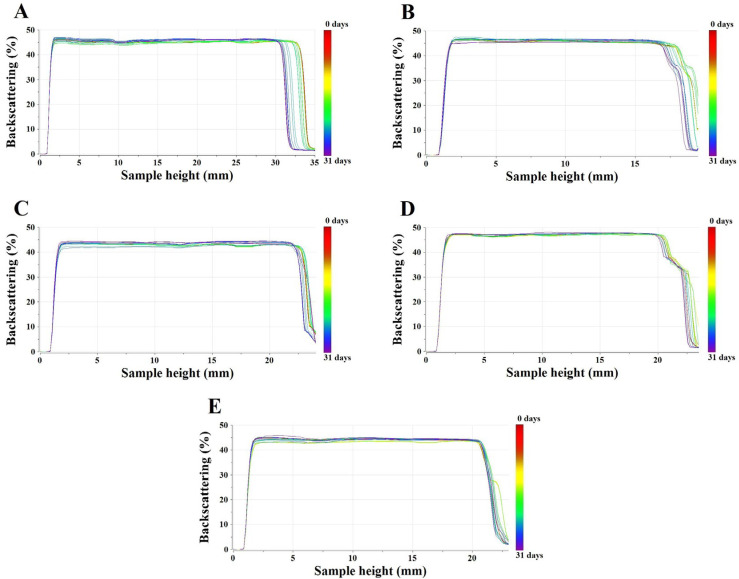
Backscattering profiles of E+[TiO_2_+SSQ1] (**A**), E+[TiO_2_+SSQ2] (**B**), E+[TiO_2_+SSQ3] (**C**), E+[TiO_2_+SSQ4] (**D**), E+[TiO_2_] (**E**).

Next, the stability of emulsions containing modified and nonmodified titanium dioxide was assessed by multiple light scattering. This method enables to detect any changes occurring in the sample without its dilution. Furthermore, it allows to shorten the aging and development processes of new formulations [37]. The changes in backscattering profiles were only considered because the samples were non-transparent and, therefore, the transmission signals were imperceptible. The sample is considered as stable when the curves overlap over time. In turn, for unstable emulsions, the changes in backscattering profiles will be detected. Two types of destabilization phenomena can be identified by multiple light scattering such as migration or particle size variation [38]. When the particles move from the bottom to the top, the creaming process occurs in the samples. In turn, when the movement of particles from the top to the bottom is observed, the sedimentation process takes place. On the other hand, when the variation in particle size occurred known as flocculation, the changes were visible in the backscattering profiles along the whole height of the samples [39]. Figure 11A–E presents the intensity of backscattered light versus sample height. The data were collected at a certain period for 31 days. The red color represents the first measurement and the blue one, the latest one. It was observed that the level of backscattering intensities for all emulsions obtained was similar and did not change significantly within 31 days. The backscattering profiles overlap over time. Only variations were observed at the top of the sample where the meniscus lowered down slightly, most likely due to an incomplete alignment. There were no significant changes in the stability over 31 days. According to Mengual et al., samples are considered as unstable when the variation of backscattering intensity exceeds 10%.

**Figure 12:**
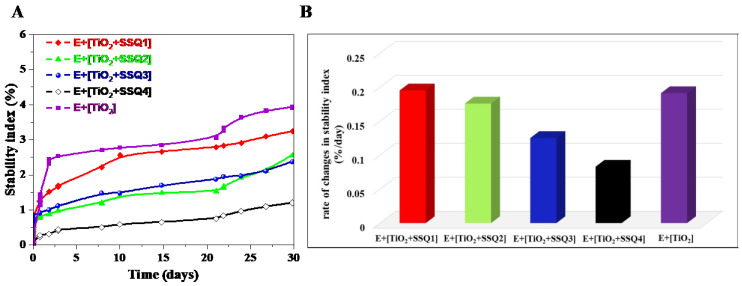
Stability indexes (**A**) and rate of changes in stability index (**B**) for emulsions E+[TiO_2_+SSQ1], E+[TiO_2_+SSQ2], E+[TiO_2_+SSQ3], E+[TiO_2_+SSQ4], E+[TiO_2_].

In order to better visualize the changes occurring in samples, the stability index was determined (Figure 12A). This parameter takes into consideration all the variations in size and concentration occurring in each sample [40]. It enables to compare the samples and select the most stable one. The lower value of the stability index is the more stable emulsion [41,42]. The value of stability index after 31 days of measurements decreased in the following order: E+[TiO_2_] (3.9%) > E+[TiO_2_+SSQ1] (3.1%) > E+[TiO_2_+SSQ2] (2.5%) > E+{TiO_2_+SSQ3] (2.4%) > E+[TiO_2_+SSQ4] (1%). Furthermore, based on the curves obtained, the rate of changes in the stability index was also determined (Figure 12B). It can be seen that the slowest variations in the stability index were observed for E+[TiO_2_+SSQ4]. Taking the above into consideration, it can be stated that emulsions containing TiO_2_ modified with the compound SSQ4 are the most stable. These results proved that the functionalization of titanium oxide increased the emulsion’s stability compared to pure titanium oxide.

This might be caused by the different nature of obtained materials that changed significantly after modification with spherosilicates derivatives (negative zeta potential) in relation with nonmodified TiO_2_ (positive zeta potential, Table 4). The results of zeta potential value may have influence on the stability of emulsions. It was observed that the highest value of zeta potential was observed for TiO_2_+SSQ4 and, thus, the emulsion containing this material was the most stable. As it was presented in Figure 9, the improvement of dispersibility of titanium dioxide by functionalization with selected spherosilicates had an influence on better stabilization of emulsions containing novel materials compared to sample E+[TiO_2_]

**Figure 13:**
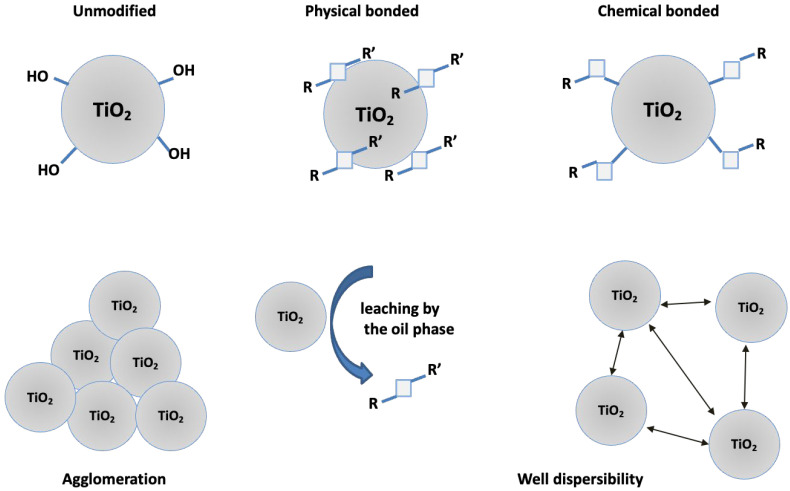
Interaction of SSQs (R/R’) with titanium dioxide in the colloidal system.

The demonstrated changes in the stability of the emulsion systems are related with two key factors such as the hydrophobic/hydrophilic properties of the modifiers and the covalent bond formed between the modifier and the hydroxyl surface of TiO_2_ via trimethoxysilyl groups in SSQ3 & SSQ4. The stability of emulsions containing modifiers that are not bound covalently to titanium dioxide such as SSQ1 and SSQ2 may be disturbed by the diffusion of the modifiers from the surface of TiO_2_ to the oil phase (Figure 13). SSQ1 and SSQ2 do not have in their structure the functional groups capable of forming covalent bonds. In turn, SSQ3 and SSQ4 are composed of a trimethoxysilyl group, which, during the deposition process, is hydrolyzed and condensed with hydroxyl groups located on the TiO_2_ surface [43].

## 4. Conclusions

In this study, hybrid systems consisting of inorganic UV filter (TiO_2_) and organosilicon derivatives (containing cinnamate or eugenol groups) with ultraviolet absorbing properties were obtained. The modification of titanium dioxide with organofunctional spherosilicates changed the nature of the materials from hydrophilic to hydrophobic. It was proved that the functionalization of titanium dioxide with spherosilicates significantly improved the dispersibility of novel materials in the colloidal system compared to nonmodified TiO_2_. Therefore, the stability of emulsions containing novel materials was also enhanced. The research showed that the key factor that had an influence on the stability of emulsions was to create a covalent bond between the modifier and the surface of titanium dioxide. The new type of hybrid inorganic–organic UV filter might be a promising agent that improves dispersibility in colloidal systems.

## Figures and Tables

**Table 1:** Substrates used to perform reactions.

Sample Name	Core	Olefin 1	Olefin 2	Olefin 3	Molar Ratios
SSQ1	octaspherosilicate	allyl cinnamate	hexene	DGME *	4:3:1
SSQ2	octaspherosilicate	allyl cinnamate	hexene	allyl glycidyl ether	3:4:1
SSQ3	octaspherosilicate	allyl cinnamate	octene	VTMOS **	4:3:1
SSQ4	octaspherosilicate	eugenol	octene	VTMOS **	3:3:2

* DGME—diethylene glycol monovinyl ether. ** VTMOS—vinyl trimethoxysilane.

**Table 2:** Composition of emulsions.

Phases	Components	Amount
	Water	64.30%
Water phase	Glycerin	7.50%
	Xanthan gum	0.30%
	Glycerol stearate	2.00%
	Polyglyceryl-3-methylglucose distearate	2.00%
Oil phase	Cetyl alcohol	3.00%
	Butyrospermum parkii butter	7.50%
	Vegetable oil	7.50%
	Modified TiO_2_/TiO_2_	5.00%
	Phenoxyethanol	0.90%

**Table 3:** Density, refractive index, and molecular weight of SSQs.

Name	Density (g/mL)	Refractive Index	Molecular Weight (g/mol)
SSQ1	1.1119 ± 0.0002	1.4934 ± 0.0002	2152.66
SSQ2	1.0953 ± 0.0002	1.4832 ± 0.0003	2030.66
SSQ3	1.1077 ± 0.0001	1.4888 ± 0.0002	2252.73
SSQ4	1.0929 ± 0.0003	1.4740 ± 0.0003	2140.70

**Table 4:** Particle size distribution and zeta potential of nonmodified and modified titanium dioxide.

Name	d (0.1)	d (0.5)	d (0.9)	Zeta Potential (mV)
[TiO_2_+SSQ1]	0.134 ± 0.004 μm	0.303 ± 0.014 μm	0.577 ± 0.020 μm	−34.2 ± 0.9
[TiO_2_+SSQ2]	0.139 ± 0.002 μm	0.307 ± 0.010 μm	0.576 ± 0.024 μm	−34.9 ± 4.3
[TiO_2_+SSQ3]	0.132 ± 0.008 μm	0.297 ± 0.007 μm	0.574 ± 0.032 μm	−35.5 ± 4.7
[TiO_2_+SSQ4]	0.134 ± 0.005 μm	0.303 ± 0.012 μm	0.579 ± 0.032 μm	−39.5 ± 0.9
[TiO_2_]	0.123 ± 0.002 μm	0.293 ± 0.009 μm	0.588 ± 0.018 μm	24.5 ± 0.7

**Table 5:** Contact angle.

Name	Contact Angle
[TiO_2_+SSQ1]	138°
[TiO_2_+SSQ2]	142°
[TiO_2_+SSQ3]	130°
[TiO_2_+SSQ4]	140°
[TiO_2_]	0°

**Table 6:** Particle size distribution for emulsions.

Name	d (0.1)	d (0.5)	d (0.9)
After Preparation			
E [TiO_2_+SSQ1]	0.961 ± 0.008 μm	1.94 ± 0.024 μm	18.0 ± 0.042 μm
E [TiO_2_+SSQ2]	0.932 ± 0.010 μm	1.78 ± 0.031 μm	9.60 ± 0.035 μm
E [TiO_2_+SSQ3]	0.973 ± 0.012 μm	2.18 ± 0.028 μm	22.7 ± 0.032 μm
E [TiO_2_+SSQ4]	0.964 ± 0.014 μm	1.92 ± 0.023 μm	11.6 ± 0.018 μm
E [TiO_2_]	0.939 ± 0.007 μm	1.76 ± 0.018 μm	8.55 ± 0.029 μm
After 3 months			
E [TiO_2_+SSQ1]	0.594 ± 0.011 μm	1.26 ± 0.017 μm	31.3 ± 0.019 μm
E [TiO_2_+SSQ2]	0.574 ± 0.008 μm	1.30 ± 0.014 μm	25.5 ± 0.038 μm
E [TiO_2_+SSQ3]	0.614 ± 0.012 μm	1.29 ± 0.026 μm	20.5 ± 0.028 μm
E [TiO_2_+SSQ4]	0.531 ± 0.009 μm	1.28 ± 0.032 μm	38.1 ± 0.030 μm
E [TiO_2_]	0.591 ± 0.013 μm	1.58 ± 0.019 μm	52.6 ± 0.045 μm

## Data Availability

All the data were collected and provided in the main manuscript.
